# The Right Ventricle: Biologic Insights and Response to Disease: Updated

**DOI:** 10.2174/157340313805076296

**Published:** 2013-02

**Authors:** Lori A Walker, Peter M Buttrick

**Affiliations:** University of Colorado Denver, Colorado, USA

**Keywords:** Right ventricle, cardiomyocyte stucture-function, physiology, pathophysiology.

## Abstract

Despite ample evidence that right ventricular function is a critical determinant of the clinical response to a spectrum of cardiovascular diseases, there has been only a limited analysis of the unique and distinguishing physiologic properties of the RV under normal circumstances and in response to pathologic insults. This knowledge deficit is increasingly acknowledged. This review highlights some of these features and underscores the fact that rational therapy in RV failure needs to acknowledge its unique physiology and ought to be chamber specific. That is proven therapies for LV dysfunction do not necessarily apply to the RV. The updated version of this review now acknowledges recent advances in the understanding of metabolic, inflammatory and gender-specific influences on the right ventricle.

## INTRODUCTION

Ever since the 17^th^ century, when Harvey first described the integrated nature of the circulation, physicians have known that right ventricular cardiac output, in the absence of intracardiac shunting, is equal to left ventricular cardiac output. Early cardiovascular investigators also recognized that the RV was structurally, geometrically and mechanically distinct from the LV, and that its response to disease states was likewise quite different. Despite this, comparatively little attention has been paid to the basic physiology and biology of the RV and the general assumption has been that the laws that define cardiac mechanics, and therapies that improve cardiac performance are equally applicable to the RV and the LV. This is only partly true. 

Given the increasingly nuanced appreciation of the cell biology of cardiac (mal)adaptation as well as increasingly sophisticated biophysical and imaging tools, a careful reexamination of the structure and function of the RV is now in order [1, 2]. Indeed, the NHLBI organized a working group in 2006 [3] in order to frame this discussion and this group identified a number of fertile areas for RV related translational research, including the distinguishing characteristics of the right and left heart, the mechanism and role of right ventricular hypertrophy, the effect of pulmonary disease on RV function, and ultimately the design of therapeutic strategies tailored to RV disease. Thus, the goals of this paper are to review the current literature as it relates to normal RV physiology and the response of the RV to pathologic circumstances. 

## I DEVELOPMENT, STRUCTURE AND FUNCTION OF THE RIGHT VENTRICLE

The early embryologic divergence in the origins of the right and left ventricles ultimately underlies the chamber-specific responses to pressure and volume overload. The left ventricle derives from the heart tube whereas the RV derives from cardiac precursors cells in the anterior heart field [4]. At the cellular level, differentiation of myoblasts into RV versus LV myocytes occurs early in fetal development. This differentiation is directed by the temporally discrete and chamber-specific expression of basic helix–loop–helix (bHLH) transcription factors. RV myocyte differentiation is driven by dHAND, whereas the development of LV myocytes is dependent on eHAND [5]. Interestingly, the interventricular septum shares its embryologic origins with the RV and not the LV. RV and LV wall thickness increase in parallel through gestation and both chambers are roughly equal in thickness at birth [6]. In utero the RV ejects blood at relatively high pressure and the nonventilated lung is largely bypassed, as blood shunts through the ductus arteriosus and foramen ovale. The postnatal circulatory transition is stimulated by active (oxygen and nitric oxide (NO) - induced vasodilatation) and passive (lung expansion), processes [7]. In the neonate the pulmonary arteries are thin-walled with an endothelial layer that is metabolically active and a single layer of smooth muscle cells that regulates vascular tone. The post-natal pulmonary circulation quickly becomes a low-pressure circuit with far lower mean pulmonary artery pressure and pulmonary vascular resistance (PVR) than is seen in the systemic circulation and, as a result, the relatively thick walled fetal right ventricle regresses leaving a thinner walled mature RV, one that is approximately a third of the thickness of the LV. How this unique embryology and unusual developmental pathway from an initially a thick walled to the more mature thin walled chamber impacts the response of the mature RV to superimposed disease is conjectural. Certainly some embryologic differences between the RV and LV appear to persist into adulthood. For example, quantitative differences in expression of key transporters and pumps are found in adult rats [8], which suggest that there are chamber selective therapeutic targets that could enhance RV function.

Anatomically the two most striking features of the right ventricle, relative to the left, is its complex shape which defies easy geometric approximation – it appears triangular when viewed from the side and crescentic when viewed in cross-section with a relatively thin free wall [9]. The muscle fibers that comprise the RV have generally been seen as forming two layers – a superficial layer arranged circumferentially and parallel to the AV groove and a deep layer that is arranged longitudinally, from apex to base, a structure that allows only a limited range of contractile motion, predominately longitudinal shortening. It is this latter plane of shortening that is described by the echocardiographic measure of RV systolic function called tricuspid annular plane systolic excursion (TAPSE). When RV shortening (systolic function) is reduced, so is TAPSE. This is in contrast to the far thicker fibers of the LV which are wrapped around the elliptical chamber in a more complex fashion – two anti-parallel muscular layers separated by a circumferential muscle band, which permits the complex motion of LV contraction, including torsion, shortening and thickening [9, 10]. The endocardial surface of the RV is generally more heavily trabeculated than the LV and a circumferential moderator band is often seen in its apical segment. A final and quite important feature of the RV relates to the anatomical structure of the tricuspid valve which has the largest annulus in the heart and is tethered by more than three papillary muscles [9, 11], features that conspire to make it a valve that is most vulnerable to structural deformation (for example in response to a sustained pressure or volume load). 

Under normal circumstances, the RV is coupled to a low pressure, highly distensible pulmonary vascular tree. Consequently right-sided pressures are lower than left-sided pressures and evidence an earlier systolic peak and more rapid pressure decline [12]. The mean RVEF for a normal individual is slightly less than that seen in the LV, and as a consequence RV volume is slightly larger, resulting in virtually identical RV and LV cardiac output. When both the pulmonary vasculature and LV are normal, RV function, though important, is not absolutely essential to support the circulation at rest, at least for the short term. Evidence supporting this includes the clinical success of the Fontan procedure that eliminates the RV from the circulation; functional capacity and cardiac output are reasonably well preserved in patients after the Fontan procedure even though total caval flow is passively directed into the pulmonary circulation. 

By virtue of the fact that the chamber thickness is far less and ventricular elastance is lower, the RV is far more afterload dependent than the LV [13, 14]. Very modest increases in pulmonary vascular resistance (one component of afterload) may result in substantial declines in RV stroke volume. This has very substantial clinical implications (see below). The preload dependence of contraction (Frank-Starling Effect) which is quite clearly manifest in the LV is similarly evident in the RV through a physiologic range of filling pressures or in response to post-extra-systolic potentiation (force-interval relationship) [15, 16], however beyond these defined margins, the functional impact of increases in RV filling are more complicated to interpret. Excessive RV filling, for example, can result in a shift in septal orientation (reverse Bernheim effect or ventricular interdependence) and LV compression with subsequent impairment of ventricular performance [16]. Moreover, the pericardium likely imposes more constraint on the thin walled, more compliant, low-pressure right ventricular chamber than it does on the left ventricle [10]. Some of the hemodynamic properties of the RV (relative to the LV) are summarized in Table 1 (derived from [2]).

Energetically, the lower systolic pressure in the RV, and hence, the lower wall stress leads to lower O_2_ requirement compared to the LV both at rest and during exercise. Consistent with the lesser external work performed by the RV, the resting RV coronary blood flow is lower than left coronary flow. Additionally, at rest, the RV extracts only ~50% of the O_2_ supplied by the coronary flow whereas the LV extracts ~75%. The lower coronary flow in the RV coupled with reduced O_2_ extraction provides both a flow reserve and a large O_2_ extraction reserve for the RV and is consistent with the clinical observation that the RV is relatively impervious to ischemic insults. In the LV, increases in O_2_ demand are primarily met by increased coronary flow [17, 18]. However, the RV can meet increases in O_2_ demands either through increases in coronary flow or by increased O_2_ extraction [17, 18]. Interestingly, exercise-induced increases in RV O_2_ demand are met primarily by increases in O_2_ extraction whereas increases in O_2_ demand induced by acute pulmonary hypertension are met primarily by increased coronary flow [19]. 

Two other related factors that impact global RV dysfunction are worth comment: loss of atrial systole and loss of synchronicity. The former has been appreciated for many years and in fact maintenance of sinus rhythm is felt to be a key therapeutic maneuver in right ventricular infarction [20, 21]. A nice experimental demonstration is from the work of Mizobuchi *et al.* [22] who demonstrated a disproportionate improvement in RV outflow track flow velocity relative to analogous measurements in the LV as a function of preserved atrial contraction. In general this phenomenon is felt to be reflective of enhanced compliance coupled with preserved length dependence of contraction. The appreciation of the importance of synchronous RV contraction is more recent and is derived from a few limited studies demonstrating that dual chamber pacing can dramatically improve RV dP/dt and cardiac index in patients with congenital heart disease, moderate RV dysfunction and right bundle branch block [23]. Whether this reflects a primary effect of the geometry of RV contraction, secondary effects on ventricular interdependence, or both is unclear. 

The regulation of RV contractility, like that of the LV, is a function of heart rate, Frank-Starling mechanisms and autonomic input. As mentioned above, within the limits of normal filling pressures, heart rate and pre-load influences on RV function are not distinct from those of the LV, although once these limits are exceeded, factors such as pericardial constraint may play a role. In so far as autonomic input is concerned, there is a differential effect on function of the inflow and outflow regions and overall adrenergic and cholinergic receptor density is generally felt to be slightly higher in the RV than in the LV [24]. Vagal input tends to prolong the normal sequence of ventricular activation beginning with contraction of the inlet and ending with contraction of the infundibulum [10], thus enhancing mechanical performance whereas beta adrenergic stimulation shortens the contraction time and may actually reverse this orderly contractile process. Some studies have suggested that alpha-adrenergic stimulation of the RV may have overall negative inotropic effects, in contrast to the well-described positive inotropy seen in the LV [25]. In addition, there are data to suggest that the summed inotropic response of the infundibulum and outflow track may be greater than that in the inflow portion of the chamber [26, 27], raising the possibility that RV cardiac output may be compromised by outflow track obstruction during periods of catecholaminergic stress, especially when the chamber is underfilled.

### Gender Differences in the Right Ventricle

It is generally well accepted that left ventricular mass and volumes differ significantly by age, sex and race [28, 29]. Attempts at defining normal mass and volumes to the RV, though, have been hampered by technical difficulties with RV imaging and estimates of RV geometry. Previous studies have shown that RV volumes are also greater in men but estimates of mass have been more variable [30-32]. More recently, in a large multicenter prospective study, it was shown that men have a greater RV mass (~8%) and a larger RV end diastolic volume than women (~10% larger), but they have a lower RV ejection fraction (by approximately 4%) [33]. These differences are similar to sex-based differences in the LV where greater mass, larger volumes and lower ejection fractions are also seen in men compared to women [28, 30]. It is thought that these differences are due, in part, to differences in testosterone, dehydroepiandrosterone (DHEA) and estradiol [34]. In this study of over 3500 men and post-menopausal women [34], higher levels of testosterone were associated with greater mass and larger RV volumes in men. Similarly, higher DHEA levels in women were associated with greater mass and larger volumes, suggesting an effect of androgens on both RV mass and volume. Higher levels of estradiol in women using hormone therapy were associated with higher right ventricular ejection fraction. This correlation between increased levels of estradiol and better RV systolic function persisted after adjustment for differences in left ventricular function suggesting the effects of estradiol on RV function are independent of effects on the LV.

### Cell Biology and Biochemical Properties of the Normal Right Ventricle 

On the cellular level, few distinctions between right ventricular cardiomyocytes and left ventricular cardiomyocytes have been described. However, there have been a number of reports showing that force generation of RV papillary muscle per unit mass is less than that of LV papillary muscle, although the shortening velocity of isolated RV muscle is greater than that of the LV [35, 36]. Additionally, isolated cell experiments comparing contractile properties of RV and LV myocytes have shown that maximal sarcomere shortening in RV myocytes was significantly less than in LV myocytes isolated from the same heart, while the diastolic sarcomere length was not different [37]. Measurements of intracellular calcium transients in isolated RV and LV myocytes show corresponding differences; that is, the peak calcium transient in LV myocytes is significantly larger than in RV myocytes, suggesting that calcium dynamics may be important in regulating the mechanical differences in these tissues. However, there are no interventricular differences in expression levels of the major calcium handling proteins in normal ventricles [37].

It is possible that the difference in contractile velocities between the RV and the LV is, in part, due to differences in myosin heavy chain isozyme expression as it has been demonstrated that there is significantly more of the α-myosin heavy chain (V1) isozyme (which is associated with a higher ATPase activity) in the RV compared to LV in both rats [35] and in rabbits [38]. It should be noted, though, that in the latter study, the Ca-ATPase activity was reduced in the RV compared to LV while shortening velocity was not measured. It is clear that the inherent differences in the extent and velocity of shortening between RV and LV cannot be explained solely by differences in myosin isozyme content and that further work is needed to sort this out.

## II RIGHT VENTRICULAR FAILURE

When clinicians and cardiovascular researchers refer to “heart failure” they are invariably describing a symptom complex that is linked to impaired left ventricular performance. This has been variably subcategorized as “ischemic” versus “dilated” and “systolic” versus “diastolic” and rarely is the function of the right ventricle commented on, except as it reflects collateral damage from left ventricular processes. This overall calculus is not completely inappropriate and in fact does reflect the incidence and prevalence of disease in western society. However it does not do justice to the facts that right ventricular dysfunction (in any setting) has profound prognostic significance and that the right ventricle evidences different biologic responses to complex pathologic loads than the LV so that therapy that is appropriate for LV dysfunction is not necessarily ideal for RV dysfunction. 

The observation that right ventricular dysfunction is a strong and independent predictor of survival in the context of LV failure dates back to the early 1980s when Polak *et al.* [39] showed that survival of patients with NYHA II-IV symptoms was strongly and inversely correlated with RV ejection fraction (<35% RV EFx was associated with a 23% 2 year survival vs 71% with a normal RV, irrespective of LV EFx). This same finding has been confirmed more recently by several other groups [40, 41] and has also been extended to show correlations between pulmonary artery pressure, RV enlargement and survival that are more strongly predictive of survival than LV EFx [42-44]. Even in patients with biopsy proven myocarditis and left ventricular dysfunction, impaired right ventricular contractile indices, such as tricuspid annular systolic excursion, are associated with a greater likelihood of death or transplantation [45]. While the converse has not been demonstrated, namely that improvement in RV function in the context of LV dysfunction positively impacts prognosis, nonetheless it would seem to follow that an appreciation of the factors that contribute to RV dysfunction and of potential chamber specific therapy, is important and timely. 

It is not our intention to comprehensively review the causes and mechanisms of right heart failure but rather to highlight some unifying principles that define the pathophysiology of RV HF. However, it is worth outlining the spectrum of diseases that impact on right ventricular function in order to provide a framework for a more mechanistic overview. This is detailed in Table 2, below, which provides a list of pathophysiologic categories rather than a comprehensive list of disease entities:

### Response of the Right Ventricle to a Pathologic Load

The right ventricular response to a pathologic load is complex and reflects the nature, severity and chronicity of the insult. In addition, the timing of the insult (during neonatal, pediatric or adult life) is important and insults that are initiated early in life (such as congenital pulmonic stenosis) tend to be better tolerated than those imposed during adulthood. This is an interesting phenomenon and likely reflects the fact that the normal RV is relatively hypertrophic during fetal and neonatal life (RV and LV wall thickness and force development are equivalent in utero) and RV hypertrophy normally regresses during infancy as it accommodates to a lower resistance pulmonary circulation [46, 47]. When confronted with a persistent increase in pressure load such as is seen with congenital pulmonic stenosis, RVH persists which may help inure the chamber against afterload-induced decompensation. However, not all causes of pressure overload are equally detrimental. The RV tolerates pulmonic stenosis considerably better than the equivalent degree of RV hypertension caused by pulmonary vascular disease. The reasons for this are not fully understood but may indicate a role for pulmonary vascular conductance that is distinct from simple mechanical overload or alternately that the RV pressure overload is best tolerated when the RV has not undergone involution (as is the case in pulmonic stenosis).

As mentioned above, the RV is generally felt to tolerate volume overload better than the LV. Mechanically this probably reflects improved muscle compliance and clinically is evidenced by the fact that RV systolic function remains well preserved even in the face of long-standing volume overload secondary to an atrial septal defect (ASD) or tricuspid regurgitation [48]. What eventually limits integrated cardiovascular function in this context is ventricular interdependence with the associated shift in the interventricular septum as well as increased pericardial constraint resulting in a reduction in LV cardiac output and diminished LV elastance, as well as the fact that over circulation of the pulmonary vasculature may eventually translate into fixed pulmonary hypertension and an increase in RV afterload [48]. 

In contrast, the adult RV appears to tolerate acute increases in afterload poorly. This was very nicely demonstrated by MacNee *et al.* [14] (among others), who showed experimentally that an acute increase in pulmonary artery pressure of 20 mmHg resulted in a 30% decline in RV stroke volume whereas an analogous increase in LV afterload resulted in only a 10% decline in LV SV. Mechanistically this likely reflects the relatively thin wall of the RV as well as reduced elastance [49]. Moderate to severe acquired pulmonary hypertension usually results in RV dilation and failure and even modest elevations in acute pulmonary vascular resistance secondary to acute pulmonary embolization can result in the inability of the RV to generate adequate systolic pressure and a precipitous fall in right ventricular stroke volume [50]. 

### Cell Biology of Right Ventricular Failure

Much of the early mechanical description of overall cardiac contractility was provided by examination of muscle preparations from the RV, since as described above, the RV is more highly trabeculated than the LV and working preparations were easier to obtain. Therefore, there is a fairly robust body of literature examining functional changes in the RV in different models of cardiac (both RV and LV) failure. However, not only is there still much controversy over the mechanism of the functional change in the failing myocyte, the nature of the functional change is still unclear. There are reports that in skinned fiber preparations from spontaneously hypertensive heart failure prone (SHHF) rats, the myofilament function (force generated at a constant level of activator calcium) of RV trabeculae is reduced [51], increased [52], or unchanged [52], compared to control, depending on the stage of disease progression. In RV trabeculae from rats subjected to large LV infarctions with associated LV failure, there is a demonstrated decrease in RV myofilament function both in trabeculae [53] and in isolated skinned RV hypertrophic myocytes [54]. Similarly, it has recently been shown in intact RV strip preparations from rats subjected to large LV infarctions, that the force generated in the presence of either isoproterenol or calcium was markedly reduced [55]. 

It is well accepted that in cardiac myocytes, calcium regulates contraction by binding to the thin filament regulatory protein troponin C which in turn causes a conformational shift in the other troponin subunits, troponin I and T, allowing myosin binding to actin, and in general, the velocity and strength of contraction are regulated by either changes in available calcium or by the calcium sensitivity of the contractile apparatus. There are undoubtedly differences in calcium handling in failing myocytes and as demonstrated by the changes in myofilament function in skinned preparations, there are likely changes in the calcium sensitivity of the myofilaments. 

In samples from the hypertrophic RV, correlations between expression levels (and/or post-translational modifications) of numerous proteins and the degree of hypertrophy have recently been described [56]. The majority of the changes described have been in metabolic and stress-related proteins and likely reflect a shift from fatty-acid metabolism to an increase in glucose metabolism. Such metabolic shifts have been well described for various models of LV hypertrophy [57, 58], but are less well described in the RV [59]. However, the proteomic changes seen in the hypertrophied RV, including a decrease in β-oxidation enzymes and an increase in glycolytic enzymes [56], would suggest that a similar shift in energy substrate utilization occurs in the hypertrophic RV as in the hypertrophic LV. The impact of these changes in substrate utilization and the possible role of a shift from mitochondrial mediated oxidative metabolism (which is energy efficient) to glycolysis (which is limiting) has been proposed for years as a possible contributor to RV dysfunction following pressure overload [60]. Recent studies using more contemporary technologies such as FDG PET scanning have documented increased glycolysis [61] as well as abnormal mitochondrial function [62] in pressure overloaded RV. These and other similar observations have introduced the promising concept that mitochondrial dysfunction might be a targetable defect in the treatment of RV pressure overload and several investigative groups have recently begun to pursue this strategy. Two approaches worth noting are the use of dichloroacetate which reduces PDH phosphorylation and improves glucose oxidation [62] in the failing RV, and the potential use of drugs like trimetazidine and ranolazine which partially inhibit fatty oxidation with a compensatory increase in glucose oxidation [63, 64]. 

An additional feature of the pressure overloaded RV is the association with autoimmune diseases, and the consistent appearance of inflammatory cells in and around pulmonary arteries and in the RV suggests an underlying inflammatory component to RV dysfunction [65-67]. Indeed pulmonary hypertension secondary to scleroderma or other types of inflammatory lung disease has a far worse prognosis and appears to impact the RV more profoundly than more conventional types of pulmonary hypertension. In a number of animal models, inflammation has been postulated to play an important role in the progression of left ventricular heart failure so the concept is not new. In some studies, a direct causal link between left ventricular dysfunction, left ventricular remodeling and expression of pro-inflammatory cytokines, including TNF-α and IL-6, has been established [68, 69]. Histologic evidence of chamber specific inflammation and mono-nuclear cell infiltration is also seen in virtually all well studied animal models of RV dysfunction, including hypoxia, monocrotaline-induced PAH [70], and sugen treatment [71]. In a more clinically relevant context, a rat heart model of pulmonary embolism, increases in chamber specific expression of CINC1, CINC2, MIP2, MCP-1 and MIP1α correlate with the severity of RV dysfunction [72]. 

### Right Ventricular Response and Rational Therapy for Pulmonary Hypertension 

While the most common cause of RV failure is LV failure, it is probably more instructive to dissect the RV response and therapeutic options available to treat RV decompensation in the setting of pulmonary hypertension. Of course many of the same principles hold for the treatment of combined RV and LV failure but the focus in this context has been on the LV. In pulmonary hypertension, the RV is primarily and predominantly affected so the physiology and therapy is more chamber specific. 

While the classification of pulmonary hypertension is extensive and the pathogenesis is complex, nonetheless the unifying feature of the disease complex is that the RV is exposed to a progressive pressure load. Variables that amplify the maladaptive response of the RV, beyond pure pressure load might include vascular impedance as well as local inflammation. While the initial adaptive response of the RV to a pressure load is myocardial hypertrophy (much like the LV when exposed to a pressure load), this is generally not adequate to normalize wall stress and progressive contractile dysfunction and chamber dilation occurs [73]. This is characterized by rising filling pressures, a decline in contractile indices, increased sphericity of the RV chamber (with likely loss of synchronous contraction), dilation of the tricuspid annulus with associated poor coaptation of the valve leaflets [13, 74, 75]. As a result, there is a functional tricuspid regurgitation and progressive volume overload of the RV. While this is functionally tolerated far better than pressure overload, the impact in this context is to amplify chamber dilation, increase ventricular wall stress, further impair indices of contractility and reduce right ventricular cardiac output. As the ventricle dilates, the ventricular interdependence becomes more pronounced and LV end-diastolic dimension decreases and left-sided stroke volume falls [76]. Whether or not global ischemia also contributes to progressive declines in contractility is probably disease dependent, but it is worth noting that in general the RV is less vulnerable to ischemia than the LV [10]. While the severity of the pulmonary artery pressure is one of the more accessible measured parameters in pulmonary hypertension, right ventricular function is the most important determinant of survival (and as RV function declines and stroke volume falls, there may be a paradoxical decline in pulmonary artery pressure) [77, 78]. Moreover, as has been mentioned previously, peak PA and/or RV pressure does not correlate with the RV dysfunction in that pulmonic stenosis is better tolerated than pulmonary vascular hypertension and inflammatory lung disease seems to result in worse RV function relative to other disease entities even at equivalent PA pressures.

Treatment of right ventricular dysfunction in this setting is largely empirical although an appreciation of right ventricular physiology would seem to support certain fundamental principles. First, since the primary insult is a pressure load with an associated increase in wall stress, a stimulus that is very poorly tolerated by the RV, afterload reduction is a primary therapy. A spectrum of vasodilators have been employed and it is quite clear that acute responsiveness to pulmonary vasodilators (regardless of class) has prognostic significance [79, 80]. Of the drugs studied, several, including endothelin receptor antagonists, such as bosentan, and phosphodiesterase 5 inhibitors, like sildenafil, appear to have independent beneficial effects on RV contractility beyond their effect on the pulmonary circulation [81, 82]. Moreover, it is clear that diminishing tricuspid regurgitation, reducing the volume load, and restoring synchronous contraction (with the secondary effect of improving ventricular interdependence) is equally important so progressive diuresis has obvious benefit. Conventional inotropes, such as dobutamine and milrinone (in normotensive RV failure) have been shown to be of use in acute right heart failure an effect that is mediated both by RV inotropy and also by virtue of their independent effect on pulmonary vascular resistance [83, 84]. Promising therapies that target distinct cellular and biochemical properties of the failing RV, such as mitochondrial dysfunction (perhaps targeted by dichloroacetate as alluded to above) and/or drugs that shift substrate utilization to more energy efficient pathways might be useful. In addition, strategies that are focused on localized fibrosis and inflammation (perhaps targeted with class specific HDAC inhibition [85] or anti-inflammatory agents (such as antibodies directed against IL-1β) may prove beneficial, although data on these strategies are as yet incomplete. 

Therapies that are of obvious benefit in LV failure, such as beta-blockade and angiotensin converting enzyme inhibition, have not demonstrated clinically significant benefit in RV failure and studies in animal models have suggested both a biologic basis for this as well as possible distinct targetable pathways. For example, Rouleau *et al.* [86] have shown, using a rabbit model of pulmonary artery banding, that RV failure results in a loss of inotropic responsiveness to Ang II and uncoupling of Ang I receptors. Studies that have suggested a benefit of beta-blocker therapy on RV function have all been done in the context of coincident LV failure so the independent impact on the RV has been difficult to assess [87]. In some models of pure RV failure, beta-blockers have proven to be deleterious, independent of their effect on pulmonary vasomotor tone although the biology underlying this is far from established [88, 89]. Fan *et al.* [89] have shown a decrease in beta-adrenergic receptor density in the RV in response to chronic pressure overload (similar to that seen in LV failure) but there are no studies that have shown that chronic beta-blocker therapy improves adrenergic responsiveness in the RV although elevated catecholamine levels may be associated with higher pulmonary vascular resistance [90]. It is also true that alpha adrenergic stimulation has differential effects on right and left ventricular trabeculae, and this biology might predict that beta-blockade would unmask the negative inotropic effects of alpha stimulation on the RV (in contrast to the positive inotropic effects on the LV) [25]. 

Given this, it is clear that the failing RV presents a qualitatively different substrate than the failing LV and pharmacotherapy should be tailored accordingly.

Maintenance of atrial systole and AV synchrony as well as synchronous RV contraction are also clearly important for the reasons articulated above and it is certainly true that progressive RV dilation secondary to a superimposed pressure load is commonly associated both with atrial arrhythmias and with the development of bundle branch block. In large series of patients with surgically corrected congenital heart disease with RV involvement, such as Tetralogy of Fallot, Epsteins’s anomaly or those following a Fontan procedure, the incidence of atrial arrhythmia is ~50% [91] and in patients with mild-moderate acquired pulmonary hypertension, the incidence is ~10-15% in retrospective studies and is estimated to occur at a rate of 2-3% per year [20, 92]. Almost all studies report that the loss of atrial systole in these contexts results in acute clinical deterioration [20, 93]. Despite this there is no consensus as to how to maintain sinus rhythm and a number of approaches, including preemptive pacing and ablative remodeling of the right atrium have been proposed. As cited above, AV sequential (DOO) pacing, when asynchronous RV contraction is demonstrated, may have the dual benefit of maintaining atrial systole and preserving synchronous RV contraction [23, 94]. 

## CONCLUSIONS

In summary, two themes emerge: the first is that the right ventricle, by virtue of its embryology, geometry and its cell biology, behaves quite differently from the left ventricle, both in normal and pathologic circumstances, and the second is that deterioration of right ventricular function strongly predicts clinical outcomes in a variety of circumstances. Thus it is imprudent to ignore the RV any longer. Understanding its physiology and developing therapeutic strategies that are chamber specific will almost certainly have broad clinical benefit.

## Figures and Tables

**Table 1.** Comparison of RV and LV Properties

Properties	RV	LV
		
EDV, mL/m^2^	75± 13(49-100)	65± 12 (44-90)
Mass, g/m^2^	26± 5 (17-34)	87± 12 (64-110)
Wall thickness, mm	2-5	7-11
Ventricular pressure, mmHg	25/4[(15-30)/(1-7)]	130/8 [(90-140)/(5-12)]
Ventricular elastance mmHg/mL	1.30± .84	5.48± 1.23
Afterload (PVR and SVR) (dyne.s.cm^-5^)	70 (20-130)	1100 (700-1600)
Accommodation to imposed load	Better in response to volume overload	Better in response to pressure overload

Abbreviations used: EDV; end-diastolic volume: PVR; pulmonary vascular resistance: SVR; systemic vascular resistence.

**Table 2.** Selected Causes of RV failure

**Pressure overload**
LV failure (most common)
Transient pulmonary processes
Pulmonary embolism
Pneumonia and other infiltrative diseases
Fixed pulmonary hypertension
Primary
Secondary (for example COPD)
Congenital disease
Pulmonic stenosis (valvular, infundibular, peripheral)
Systemic RV
**Volume overload**
Valvular insufficiency
Tricuspid, either primary or acquired
Pulmonic
Congenital disease
Atrial septal defect
Anomalous pulmonary venous return
Arteriovenous (AV) fistula
**Intrinsic muscle disease**
RV ischemia and/or infarction
Infiltrative cardiomyopathy
Amyloid
Sarcoid
ARVD
**Impaired RV filling**
Constrictive pericardial disease
Tricuspid stenosis
